# Clinical efficacy of refined nursing strategies on early rehabilitation training for postoperative patients with cervical spondylosis

**DOI:** 10.1097/MD.0000000000038127

**Published:** 2024-05-31

**Authors:** Pinyun Cai, Lijun Wu, Liqun Dai, Qingqing Yan, Qing Lan

**Affiliations:** a The First Affiliated Hospital of Fujian Medical University Binhai District National Regional Medical Center Orthopedics and Hand Surgery Ward I Fujian, China; b The First Affiliated Hospital of Fujian Medical University Spine surgery Fujian, China; c The First Affiliated Hospital of Fujian Medical University Bone tumor joint surgery Fujian, China.

**Keywords:** cervical spondylosis, life quality, nursing satisfaction, postoperative outcomes, refined nursing strategies, rehabilitation

## Abstract

To investigate the effects of refined nursing strategies on postoperative quality of life, pain level, psychological condition, and other rehabilitation training of patients with cervical spondylosis. This study is a retrospective study, and 500 patients with cervical spondylosis admitted to our hospital from April 2022 to June 2023 were selected as the study objects. They were divided into a control group (250 cases) and an experimental group (250 cases) according to whether they received personalized intensive care. The control group received standard nursing care, while the experimental group was given personalized, high-quality nursing care. The refined nursing strategy comprises individualized rehabilitation training plans, psychological support, nutritional counseling, and pain management. Indicators such as pain level, cervical spine function, quality of life, complication statistics, and nursing satisfaction were employed to evaluate the clinical efficacy of 2 nursing methods. In the pain assessment, patients in the experimental and control groups had pain scores of (1.98 ± 0.84) and (5.78 ± 0.63), respectively, after the nursing intervention. The reduction in pain for patients in the experimental group was highly significant (*P* < .05) compared to pre-nursing care. Six months after the commencement of healthcare, the cervical spine function scores of the experimental and control groups were (93.36 ± 4.74) and (82.68 ± 5.42), respectively. Moreover, the cervical spine function recovery of the experimental group was deemed significant. The experimental group exhibited a lower probability of complications compared to the control group. Additionally, the quality of life was significantly higher in the experimental group than in the control group (*P* < .05). Improvement time and healing time were significantly shorter in the patients of the experimental group in comparison to the control group. In terms of nursing satisfaction, the experimental group had a significantly higher satisfaction rate of 87.57% than the control group (*P* < .05). The refined nursing strategy significantly improved the speed and quality of patients’ functional recovery. Additionally, the implementation of personalized and comprehensive nursing techniques during postoperative treatment for cervical spondylosis resulted in a significant improvement in patient’s quality of life and satisfaction with the treatment process.

## 1. Introduction

With the accelerated pace of modern life and increased work pressure, cervical spondylosis (CS) has become a common health problem that seriously affects people’s daily life and work efficiency.^[1,2]^ Meanwhile, the incidence of CS, as a prevalent chronic degenerative disease, has been increasing in recent years. CS not only causes great inconvenience to the patient’s life, but also may cause severe neurological dysfunction due to the compression of the nerve roots or spinal cord.^[3,4]^ Currently, there are various treatment modalities for CS, among which surgical treatment is widely used for its rapid and effective symptom relief. However, early postoperative rehabilitation is crucial to ensure the recovery of patients, so how to conduct postoperative rehabilitation training for CS patients has gradually become a major issue in the medical field.^[5]^ Traditional postoperative care often uses standardized procedures, a one-size-fits-all approach that ignores individual patient differences and may lead to poor recovery.^[6]^ In recent years, personalized nursing strategies and refined nursing strategies (RNS) have gradually gained attention. Refined care considers not just the patient’s physiological state, but also incorporates their psychological, social, and cultural background to enhance their recovery in a more comprehensive approach.^[7,8]^ The superiority of RNS lies in its relevance and flexibility. Individualized rehabilitation plans tailored to each patient’s specific condition and needs can more effectively promote postoperative recovery and reduce complications.^[9]^ In addition, fine-tuned care also includes psychological support and social adaptation guidance for patients, which helps to improve their overall recovery.^[10]^ Based on this background, the study sought to examine the impacts of RNS on the life quality (LQ), pain management, psychological condition, and nursing satisfaction (NS) of patients with CS. Through the experiment, it is expected to provide a more comprehensive and effective treatment program for the early recovery of postoperative CS patients, thus making a substantial contribution in improving patients’ LQ.

The innovation of the study lies in the application of RNS to early rehabilitation training after CS. Through a personalized rehabilitation program, it aims to improve the rehabilitation efficiency and LQ of patients. The study not only focuses on the physical recovery during rehabilitation but also looks at the psychological health and social adaptability of the patients, to achieve a more comprehensive rehabilitation effect.

## 2. Information and methodology

### 2.1. General information

This study is a retrospective study, and 500 patients with CS admitted to our hospital from April 2022 to June 2023 were selected as the study objects. They were divided into a control group (250 cases) and an experimental group (250 cases) according to whether they received personalized intensive care. The patients and their families willingly completed an informed consent form, and the study was authorized by the hospital’s Medical Ethics Committee. Inclusion criteria: all of them met the international diagnostic criteria for CS; the patients’ ages ranged from 40 to 60 years old; all of them were diagnosed with CS after X-ray and CT laboratory imaging; all of them underwent surgery for CS; the patients were conscious and able to communicate normally and carry out free activities; all of them participated voluntarily and signed an informed consent form to carry out the experimental observation normally; and the patients’ s were all educated in junior high school and above. Exclusion criteria: patients with severe confusion; women during lactation or childbirth; patients with fractures, frozen shoulder, bone tumors, etc., and other malignant diseases; diagnosis accompanied by other symptoms of different types of CS; those with renal failure; patients with poor compliance; and comorbidities with other malignant diseases.

### 2.2. Research methods

#### 2.2.1. Control group

The following standard nursing care was provided to the patients in the Control group (CG): Keeping the environment clean: Managing the environment of the ward, ensuring that the postoperative rooms are sunny, with appropriate temperature and humidity, away from noise, and keeping the environment clean and tidy. Monitoring signs: Monitoring the clinical signs of the postoperative patients, and providing guidance and advice on the use of medication and diet. Improve the examination and carry out activities: improve the postoperative examination of patients, organize some CS postoperative health education activities, and through the relevant activities, guide and assist the patients to carry out the rehabilitation training. Drug administration: As directed by the physician, ensure that patients take the appropriate therapeutic medications in the prescribed dosage and at the appropriate times. Nutritional advice: Encourage patients to eat more meals high in protein, vitamins, and trace elements while also taking precautions with their diets. Wound cleaning: Take regular care of the postoperative wounds to reduce the risk of wound infection.

#### 2.2.2. Experimental group

Refined nursing care was provided to the patients in the Experiment group (EG); individualized and refined nursing care measures were added after the nursing care of the CG served as a basis: Improving the quality of nursing care: Through the establishment of a professional training system, nursing staff are regularly trained in theoretical and practical skills. Strengthening the assessment mechanism and rewarding nursing staff with excellent performance in order to enhance their sense of responsibility and ability to provide quality nursing services. Psychological state monitoring: Considering the pain and inconvenience caused by the symptoms of the disease and the special nature of the surgical site, patients may feel fearful of CS surgery. Nurses should patiently explain the process and purpose of the surgery, relieve patients’ worries and anxiety, and inform patients of relevant cases of successful clinical treatments in order to enhance their confidence in the success of the surgery. Preoperative preparation and training: During and after cervical spine surgery, patients may have to stay in a flat position all the time for bed rest because of the surgery, so they can be trained to push the trachea and get used to the daily life and activities in bed before the surgery to adapt to the postoperative sedentary life. Postoperative observation and nursing care: After surgery, nursing staff should closely monitor the healing of the surgical site and the patient’s breathing. Since cervical spine surgery may affect the patient’s respiratory airway and cause respiratory obstruction, it’s important to monitor the patient’s breathing rate, respiratory secretions, drainage status, and laryngeal swelling, and pay attention to the patient’s face color to see if there is any abnormality. In the event of adverse symptoms, the doctor should be notified and assisted immediately. Develop personalized rehabilitation training: Implement rehabilitation training from the first day after surgery, focusing on muscle strength and body coordination training. Various methods are used to relieve patients’ pain, such as attention diversion and soothing music. Encouragement and praise are given to patients who continue to train. Tools such as exercise balls and piercing needles were utilized to improve hand dexterity, and lower limb and back training was instructed. Combine acupuncture, physical therapy, and massage to promote blood circulation. Give rewards and appreciation to patients who complete the training tasks to enhance their confidence in rehabilitation. Discharge guidance: When the patient’s symptoms are stabilized, he or she can be discharged, but the nursing staff must inform the patient and his or her family about how to protect the neck after discharge, suggesting that he or she wear a neck brace when going out at the initial stage and that he or she go to the hospital for regular postoperative checkups.

### 2.3. Observation indicators

The following 7 markers in total were used to assess the patients’ postoperative state: Pain assessment: The degree of pain in both groups was measured using a visual analog scale. The scores were on a scale from 1 to 10. The more acute the pain, the higher the score. Cervical spine function assessment: The Japanese Orthopaedic Association Assessment was applied to assess the specific recovery of cervical spine function in the 2 groups. The cervical spine’s range of motion was graded up to a maximum of 20, with higher scores denoting a more complete recovery for the patient. A full score of 100 was assigned to the cervical spine’s function; a higher and larger score denoted a more favorable outcome for the patient. LQ assessment: Using the Short Form of Health Status Survey, which has a maximum score of 100, the quality of survival of patients in the 2 groups was evaluated. The assessment covered somatic and social functions, general health status, physical and emotional functions, etc. Anxiety and depression are compared: Patients’ anxiety was measured using the Self-Rating Anxiety Scale (SAS). Furthermore, the patients’ depressed mood was evaluated using the Self-rating Depression Scale (SDS). Both measures had a total score of 100; the lower the patient’s psychological state, the higher the score. Recording of clinical indicators: recording and comparing the operation time, average postoperative hospitalization days, and hospitalization costs of the 2 groups of patients. Complication statistics: Data on the 2 groups’ incidence of various problems, such as dysphagia, hypotension, thirst, hunger, nausea, and urine retention. NS survey: The patient’s satisfaction was counted in an anonymous way, with a full score of 100 points, which can be divided into satisfaction (higher than 80 points), general satisfaction (60–79 points interval), and dissatisfaction (less than 60 points). ns = (number of satisfied + number of generally satisfied)/total number × 100%.

### 2.4. Experimental data processing and analysis

Data analysis was conducted using SPSS 26.0. Measurement data were reported as mean ± standard deviation ($\bar{x}\pm s$) using t-test, while count data, such as disease kind, were expressed as percentage using $\chi^{2}$ test. A statistically significant change is indicated if *P* < .05.^[11]^

## 3. Results

### 3.1. Analysis of general information

A study was conducted on five hundred post-CS patients who were admitted to the hospital between January 2021 and December 2022. The patients were distributed in age groups of 50 to 60 years old. Table 1 displays the basic data for the patients taking part in the study. The average age of the 125 male and female patients in the EG was found to be (56.24 ± 5.24) years. There were 120 female and 130 male patients in the CG, with an average age of 58.63 ± 6.32 years.

**Table 1:** Basic data of experimental patients.

Basic indicators		Experimental group (n = 250)	Control group (n = 250)	*P*	_ $\chi^{2}/t$ _
Age		56.24 ± 5.24	58.63 ± 6.32	.252	3.244
Male		125	130	.224	3.156
Female		125	120	.326	2.012
Patient weight (kg)		62.65 ± 2.13	61.35 ± 3.14	.111	1.358
Patient height (cm)		162.21 ± 4.87	161.36 ± 5.63	.320	1.230
Disease type	Spinal type	107	136	.558	–
	Radicular type	143	114	.275	
Smoking	Yes	116	117	.016	
	No	134	133		
Blood sugar	Yes	128	128	.000	
	No	122	122		
Education level	Junior high school and high school	129	138	–	
	High school or above	121	112		
Hypertension (examples, %)		82	94	.048	
Diabetes (example, %)		44	52	.032	
Coronary heart disease (cases, %)		52	40	.177	

### 3.2. Comparison of pain scores between the 2 groups

The comparison of the 2 groups’ pain scores is displayed in Table 2. The experimental group’s pain score was 8.79 ± 1.17 before care, while the CGs’ score was 8.85 ± 1.21 before care. Following treatment, the EG and CG had pain scores of 1.98 ± 0.84 and 5.78 ± 0.63, in that order. When the 2 groups were compared, it was clear that the EG was significantly reducing the amount of pain.

**Table 2:** Comparison of pain scores between the 2 groups of patients.

Group	Number of examples	Before care	After care	*t*	*P*
Control group	250	8.85 ± 1.21	5.78 ± 0.63	3.976	<.05
Experimental group		8.79 ± 1.17	1.98 ± 0.84*	3.162	<0.05
*t*	–	0.835	2.802	–	
*P*		.282	.005		

Indicates comparisons with the same group before care, “*” indicates *P* < .0.5 (below).

### 3.3. Comparison of cervical spine mobility and function scores

The cervical spine mobility ratings of the 2 patient groups are compared in Table 3. Following the nursing intervention, the left and right flexion mobility scores in the CG and EG were (15.23 ± 1.07) and (18.81 + 1.09), respectively. The initial scores for left and right flexion mobility were (12.02 ± 0.42) and (12.05 ± 0.34), respectively. This suggests that there was a significant difference (*P* < .05) in the left and right cervical spine flexion mobility between the 2 patient groups. Similar to this, it was found that there was a substantial (*P* < .05) difference in the 2 patient groups’ left and right rotation, forward flexion, and backward extension mobility, and that the CG’s mobility was significantly less than the EG’s.

**Table 3:** Comparison of cervical spine mobility scores between 2 groups of patients.

Group	Left flexion		Right flexion		Anterior flexion	
	Before care	After care	Before care	After care	Before care	After care
Control group	12.02 ± 0.42	15.23 ± 1.07*	12.21 ± 0.89	13.67 ± 6.31*	12.17 ± 0.41	16.07 ± 6.31*
Experimental group	12.05 ± 0.34	18.81 + 1.09*	12.18 ± 0.91	19.97 ± 6.32*	12.89 ± 0.54	18.85 ± 6.29*
*t*	0.044	2.136	0.025	2.247	0.254	1.258
*P*	.881	.019	.103	.001	.764	.010

Group	Sinistral rotation		Dextral rotation		Backward extension	
	Before care	After care	Before care	After care	Before care	After care
Control group	10.46 ± 0.54	12.73 ± 0.52*	13.28 ± 1.09	15.75 ± 0.65*	11.13 ± 0.33	15.19 ± 1.12*
Experimental group	9.48 ± 1.43	13.86 ± 0.63*	12.34 ± 1.15	19.46 ± 0.89*	12.28 ± 0.71	17.25 ± 1.13*
*t*	0.022	2.855	0.048	2.420	0.894	2.112
*P*	.781	.006	.894	.034	.415	.005

“*” indicates *P* < .0.5.

The comparison of the 2 groups’ cervical spine function scores (CSFS) is displayed in Table 4. It can be found that the CSFS of the EG rose very rapidly with the passage of nursing time. The CSFS of the EG and CG were (93.36 ± 4.74) and (82.68 ± 5.42), respectively, when the nursing time reached 6 months. A comparison between the 2 groups’ CSFS showed a significant difference (*P* < .05).

**Table 4:** Examining the differences in cervical spine function ratings between both patient groups.

Group	Number of cases	Before care	After 1 mo of care	After 3 mo of care	After 6 mo of care
Control group	250	29.17 ± 3.65	78.84 ± 6.23*	89.45 ± 3.47*	93.36 ± 4.74*
Experimental group		29.89 ± 3.83	54.47 ± 5.88	80.28 ± 3.29	82.68 ± 5.42
*t*	–	0.985	3.549	4.867	5.221
*P*		.432	.003	.003	.003

“*” indicates *P* < .05.

### 3.4. Comparison of the 2 groups’ quality of survival scores

The comparison of the 2 groups’ quality of survival scores is displayed in Table 5. Following the end of nursing care, the EG’s scores significantly improved over the CG’s.

**Table 5:** Comparison of quality of life scores between the 2 groups of patients.

Group	Number of cases	SF-36		*t*	*P*
		Before care	After care		
Control group	250	53.87 ± 3.82	83.28 ± 5.63*	3.876	.024
Experimental group		53.31 ± 3.76	73.24 ± 4.65	2901	.003
*t*	–	0.978	3.876	–	
*P*		.293	.003		

SF-36 = Short Form of Health Status Survey.

“*” indicates *P* < .05.

### 3.5. Statistics on the occurrence of complications

**Figure 1.**
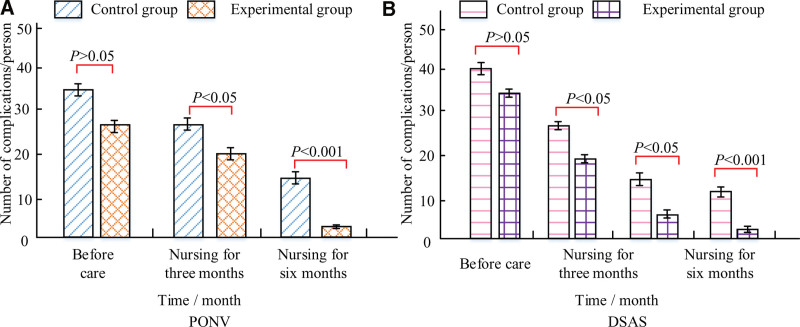
Postoperative complications of patients.

The incidence of post-operative nausea and vomiting (PONV) and difficulty swallowing after surgery (DSAS) were examined as research variables in the current investigation. Figure 1 displays the 2 groups’ complication statistics. Over time, there has been a declining trend in the number of patients in both groups experiencing PONV and DSAS problems. In the CG and EG, the number of individuals who experienced the PONV phenomenon was 16 and 4, respectively, when the nursing time reached 6 months. In the 2 groups, there were 14 versus 3 individuals who experienced DSAS symptoms, respectively.

### 3.6. Comparison of psychological status scores of patients in 2 groups

In particular, the SDS and SAS scores of the EG and CG in the first, third, and sixth months of nursing care are shown in Table 6, which compares the psychological state scores of the 2 patient groups before and after the nursing intervention. The EG’s SDS and SAS scores at 6 months of care were (8.80 ± 3.66) and (10.20 ± 10.46), respectively. On the other hand, the 2 scores on the CG were, respectively, 16.41 ± 2.90 and 15.97 ± 10.87. After treatment, the EG demonstrated a significantly lower level of anxiety and depressed symptoms than the CG (Table 6).

**Table 6:** Comparison of SDS and SAS scores before and after nursing care.

Group	Time	Pre-nursing SAS	Post-care SAS	Pre-nursing SDS	Post-care SDS
Experimental group	1	72.46 ± 3.36	56.91 ± 3.50	63.17 ± 11.34	49.60 ± 9.26
Control group	1	75.63 ± 3.42	57.20 ± 3.86	65.14 ± 11.52	51.97 ± 10.23
Experimental group	3	52.46 ± 3.36	32.81 ± 3.10	41.17 ± 11.38	30.80 ± 12.32
Control group	3	52.66 ± 3.42	33.10 ± 3.76	40.14 ± 10.06	32.67 ± 11.17
Experimental group	6	48.42 ± 3.36	8.80 ± 3.66	29.17 ± 11.21	10.20 ± 10.46
Control group	6	47.61 ± 3.42	16.41 ± 2.90	32.14 ± 11.55	15.97 ± 10.87

SAS = Self-Rating Anxiety Scale, SDS = Self-rating Depression Scale.

### 3.7. Average days of hospitalization vs. cost of hospitalization

**Figure 2.**
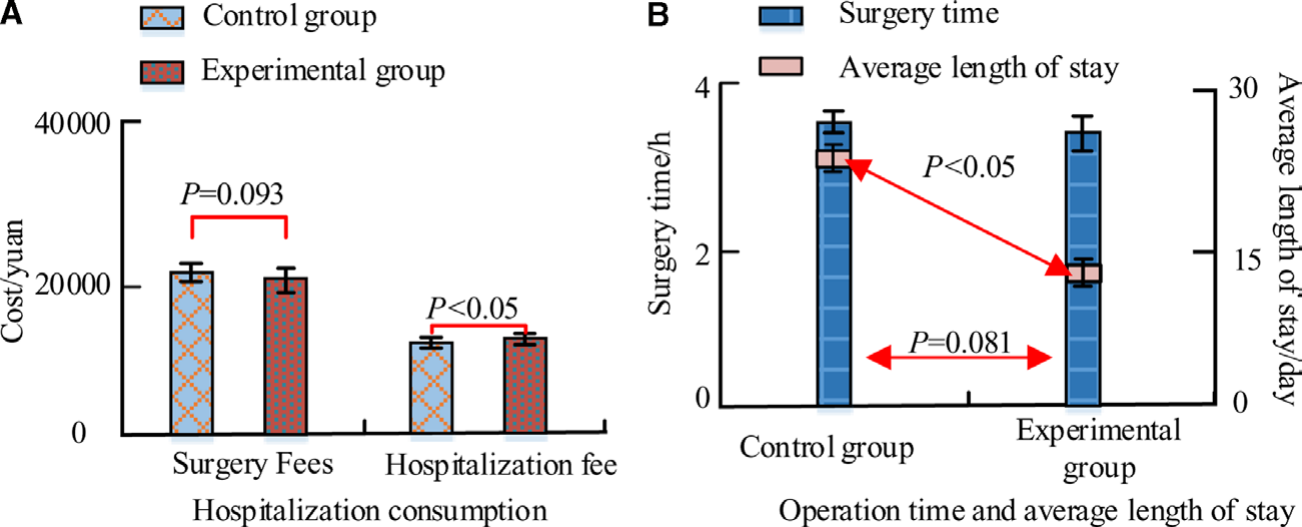
Comparison of hospitalization expenses and average length of stay between the 2 groups of patients.

Figure 2 shows the comparison between the hospitalization cost and the average number of days of hospitalization in the 2 groups. The outcomes in terms of surgery expenses and hospitalization expenditures are displayed in Figure 2A. The data showed that in the comparison of surgical cost, the CG was (22,245.21 ± 341.53) dollars. The EG was (21,403.37 ± 351.26) dollars. The costs did not differ statistically significantly (*t* = 5.41, *P* = .093). In the meantime, the CG hospitalization cost (15,236.05 ± 236.85) Yuan. The EG amounted to (16,538.59 ± 245.12) USD. Figure 2B shows how the 2 groups’ average hospital stays and operation times compare. In terms of operating time comparison, the CG required (3.86 ± 0.62) hours. It took the EG 3.83 ± 0.71 hours. In the meantime, the CG’s average hospital stay was (46.52 ± 21.26) days. In the EG, the average number of hospital days was 15.77 ± 9.12 days. The hospitalization durations of the 2 groups differed significantly (*t* = −5.27, *P* < .001).

### 3.8. Comparison of patient time to improvement and time to cure after nursing intervention

**Figure 3.**
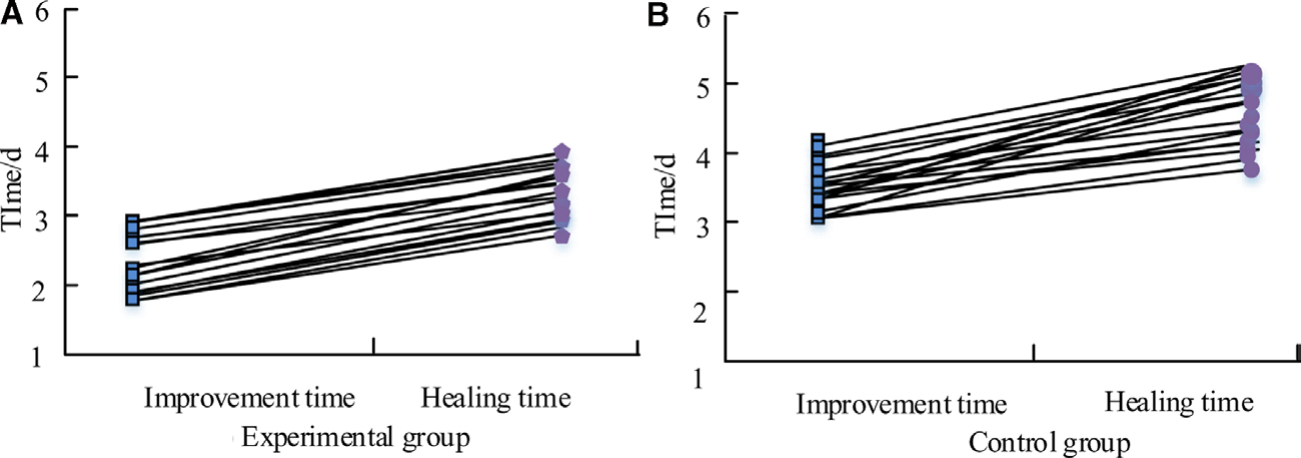
Comparison of symptom improvement and recovery time between the 2 groups of patients.

The consumption of postoperative improvement time and cure time of different patients can directly reflect the existence of differences between the two means of care, so the experiment was conducted to statistically compare the postoperative improvement time and cure time. The comparison of the 2 patient groups’ postoperative progress and cure time consumption is depicted in Figure 3. The comparison of the EG’s improvement and cure times is displayed in Figure 3A. It can be found that the EG began to improve the adverse symptoms after (2.35 ± 1.23) days, and began to tend to the healing state after (3.78 ± 1.56) days. Figure 3B shows the comparison of time consuming for improvement and time consuming for cure in CG. In the CG, the adverse symptoms started to improve at (3.47 ± 1.08) days and after (5.46 ± 1.67) days it started to converge to the cured state. There was statistical significance (*P* < .05) in the number of days of symptomatic improvement between the 2 patient groups. When comparing the 2 patient groups’ recovery times, a statistically significant difference was seen (*P* < .001).

### 3.9. Survey of nursing satisfaction

**Figure 4.**
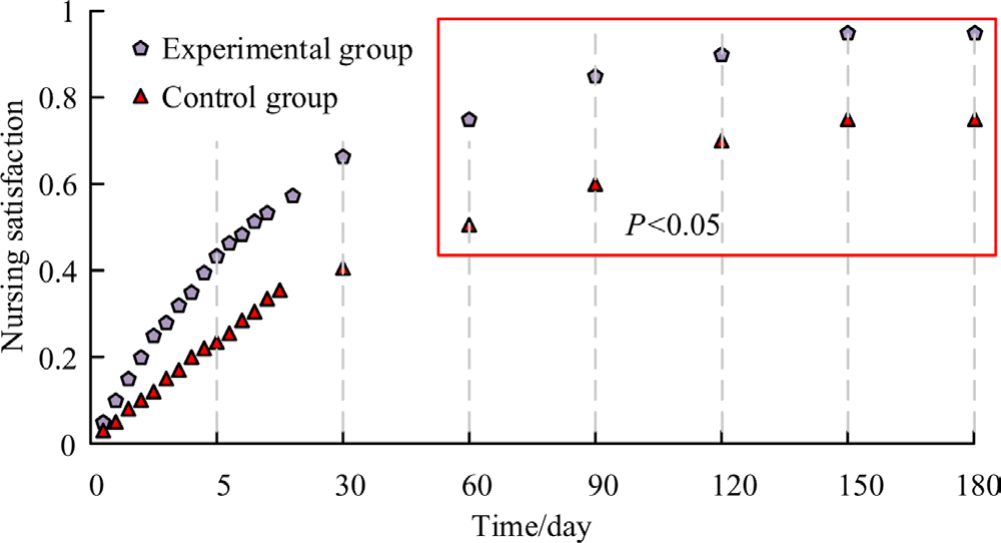
Comparison of nursing satisfaction between the 2 groups of patients.

The NS of the 2 patient groups are compared in the study at various time intervals. Comparative data can be used to determine how patients specifically tolerate various study approaches. Figure 4 displays the comparison’s findings. From day zero, when the nursing intervention started, to day 180 of the observation procedure, it was discovered that the patients in the EG were continuously happier with the methods of care than the CG. The NS of the EG achieved a maximum of 87.57% when the nursing period reached the ninetieth day. The NS of the CG was just 61.02% at this point.

## 4. Discussion

The incidence of CS is increasing due to lifestyle changes, dietary habits, and life stress, resulting in a significant impact on patients’ health and a decline in their quality of life.^[12,13]^ CS causes physical discomfort and affects patients’ daily functioning and work efficiency. While surgery can effectively relieve symptoms, patients’ complete restoration depends on early postoperative rehabilitation.^[14,15]^ During the postoperative stage of patients’ rehabilitation treatment, low self-care abilities often lead to a decline in daily activity levels due to a lack of disease and treatment-related knowledge. This can increase the risk of recurrence. Therefore, it is highly important to promote patients’ recovery and enhance their quality of life by aiding them to develop confidence in rehabilitation and bolster their psychological resilience.^[16]^ However, improving patient recovery through effective postoperative rehabilitation methods is a significant and worthwhile topic to consider. The conventional nursing model is limited in its scope and struggles to meet the diverse nursing requirements of patients. Personalized nursing interventions can assist patients in developing positive behaviors and receiving appropriate rewards and affirmations. These interventions aim to reinforce patients’ cognitive abilities and promote their confidence in recovery, ultimately supporting their overall recovery process.^[17]^ Refined care offers multiple advantages, particularly in its comprehensive and individualized approach. It takes into account the patient’s psychological and social welfare in addition to their physical recuperation. Thus, this study’s main goal was to investigate how RNS affects CS patients’ pain assessment, psychological state management, LQ, and NS.

In this study, a total of 500 postoperative CS patients were chosen, with 250 patients in each group, and split into an EG and a CG using a random number selection technique. Throughout the coherent experimental process, all patients complied with the principle of nativity. When the basic data findings were compared, it was found that there was no significant difference (*P* > .05) in terms of gender, age, education, or disease state between the 2 patient groups. Additionally, the subjects that were chosen were similar. Before the initiation of therapy, the experimental and control groups’ pain scores were (8.79 ± 1.17) and (8.85 ± 1.21), respectively. Following nursing care, the EG and CG had pain scores of 1.98 ± 0.84 and 5.78 ± 0.63, respectively. This is because RNS can very comprehensively analyze the postoperative pain situation of patients and take effective countermeasures to enhance the mental toughness of patients through psychological interventions and other methods to alleviate the pain situation of the patient’s incision. In addition, through attention transfer and other measures to reduce the patient’s sensitivity to pain and improve pain symptoms.^[18]^ Following the intervention, the CG and EG’s left flexion and right flexion mobility scores were, however, 15.23 ± 1.07 and 18.81 + 1.09, respectively. Furthermore, at the 6-month nursing time point, the EG and CG’s CSFS were (93.36 ± 4.74) and (82.68 ± 5.42), respectively. Additionally, there was a significant difference (*P* < .05) in the improvement of the survival governance ratings of the patients in both groups following the conclusion of nursing care. This indicates that personalized rehabilitation training in RNS has a very significant positive effect on restoring cervical spine function and improving patients’ LQ. The main reason is that under RNS, caregivers can follow up on the patients’ cervical spine function exercises by phone or offline visits, fully understand the patient’s condition, give timely targeted guidance, and then increase cervical spine mobility.^[19]^

In the comparison of complication statistics, the number of people who developed PONV phenomenon in the CG and the EG was 16 versus 4, respectively, when the duration of care reached 6 months. The number of DSAS symptoms in the 2 groups was 14 and 3, respectively. Both groups were highly significantly different from each other (*P* < .001). According to psychological condition scores, after receiving care for 6 months, the EG’s SAS and SDS scores were (8.80 ± 3.66) and (10.20 ± 10.46), respectively. On the other hand, the CG’s SAS and SDS scores were, respectively, 16.41 ± 2.90 and 15.97 ± 10.87. The main reason is that RNS focuses more and more on the changes in postoperative patients’ psychological status, positively intervenes in patients’ bad state of mind through a series of preaching activities, positively guides patients to live positively, and avoids the emergence of negative emotions.^[20]^ The 2 groups’ methods of care differed significantly from one another. The EG experienced a much lower average number of hospital days than the CG. NS was substantially higher in the EG at all times of the nursing intervention. The NS of the EG reached a maximum of 87.57% (*P* < .05) when the nursing time reached the 90th day.

In conclusion, RNS has demonstrated significant clinical efficacy in early post-CS patient rehabilitation. A personalized rehabilitation program can promote patients’ physical and psychological recovery more effectively, improving LQ. This finding offers new perspectives and methods for post-CS patient rehabilitation and serves as a valuable reference for future healthcare research and practice.

## Author contributions

**Conceptualization:** Pinyun Cai, Lijun Wu, Liqun Dai, Qingqing Yan, Qing Lan.

**Data curation:** Pinyun Cai, Lijun Wu, Liqun Dai, Qing Lan.

**Formal analysis:** Qing Lan.

**Funding acquisition:** Lijun Wu, Liqun Dai.

**Investigation:** Pinyun Cai, Lijun Wu, Liqun Dai, Qingqing Yan, Qing Lan.

**Methodology:** Pinyun Cai, Lijun Wu, Liqun Dai, Qingqing Yan, Qing Lan.

**Validation:** Liqun Dai, Qingqing Yan.

**Visualization:** Qingqing Yan.

**Writing – original draft:** Pinyun Cai, Lijun Wu.

**Writing – review & editing:** Pinyun Cai, Lijun Wu, Qingqing Yan.
